# Visualization of integrin molecules by fluorescence imaging and techniques

**DOI:** 10.32604/biocell.2021.014338

**Published:** 2021-02-19

**Authors:** Chen CAI, Hao SUN, Liang HU, Zhichao FAN

**Affiliations:** 1 Department of Immunology, School of Medicine, UConn Health, Farmington, 06030, USA; 2 Department of Medicine, University of California, San Diego, La Jolla, 92093, USA; 3 Cardiovascular Institute of Zhengzhou University, Department of Cardiology, The First Affiliated Hospital of Zhengzhou University, Zhengzhou, 450051, China

**Keywords:** Integrins, Fluorescence imaging, Fluorescence labeling, Live-cell imaging, Super-resolution imaging, Intravital imaging, FRET

## Abstract

Integrin molecules are transmembrane αβ heterodimers involved in cell adhesion, trafficking, and signaling. Upon activation, integrins undergo dynamic conformational changes that regulate their affinity to ligands. The physiological functions and activation mechanisms of integrins have been heavily discussed in previous studies and reviews, but the fluorescence imaging techniques -which are powerful tools for biological studies- have not. Here we review the fluorescence labeling methods, imaging techniques, as well as Förster resonance energy transfer assays used to study integrin expression, localization, activation, and functions.

## Introduction

Integrins are a family of adhesion receptors that are abundantly expressed in all cell types of metazoans except for erythrocytes. Their integral roles in mediating cell-cell and cell-extracellular matrix (ECM) interactions make integrins indispensable for the existence of multicellular organisms. Interactions between integrins and their ligands trigger profound changes of the cytoskeleton and signaling apparatus during biological processes, such as adhesion (Evans *et al*., 2019; Fan *et al*., 2019; Fan *et al*., 2016; Stubb *et al*., 2019; Sun *et al*., 2020a; Valencia-Gallardo *et al*., 2019), migration (Bernadskaya *et al*., 2019; Martens *et al*., 2020; Sun *et al*., 2014), proliferation (Clark *et al*., 2020; Erusappan *et al*., 2019), differentiation (Martins Cavaco *et al.*, 2018; Schumacher *et al*., 2020; Xie *et al*., 2019), inflammation (Arnaout, 2016; Sun *et al*., 2020b), tumor invasion (Bui *et al*., 2019; Haeger *et al*., 2020), and metastasis (Fuentes *et al*., 2020; Howe *et al*., 2020; Osmani *et al*., 2019). Fine-tuned integrin signaling is crucial for cellular homeostasis, and abnormal integrin activities give rise to many pathological conditions, including autoimmune diseases, cardiovascular diseases, and cancer. Extensive efforts have been made to discover and develop molecules targeting integrins as potential means of therapy (Ley *et al*., 2016). Several integrin-targeting antibodies and synthetic compounds are approved for treating inflammatory diseases or are under investigation in clinical trials. Fluorescent imaging techniques provide a powerful tool for better understanding integrin structures and conformational changes (by Förster resonance energy transfer, conformational reporting antibody, and super-resolution imaging), and integrin-ligand interactions to develop more effective therapies for a vast array of diseases.

### Structure of integrins

**FIGURE 1.**
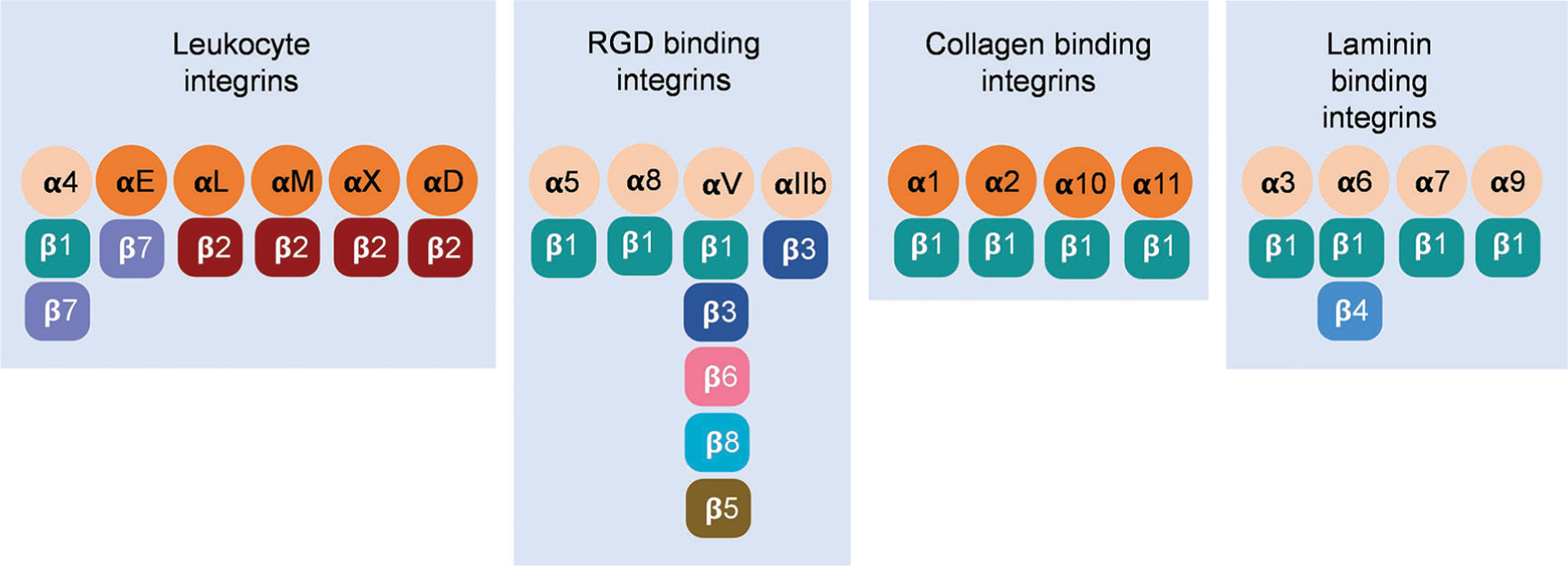
Twenty-four αβ pairs of vertebrate integrins constituted by 18 α subunits and 8 β subunits have been classified into four separate groups. Dark and light oranges represent α subunits with or without the αA/αI domain. Different β subunits were colored differently. RGD is the abbreviation of Arg-Gly-Asp peptides.

Integrins are heterodimers consisting of noncovalently associated α (120–180 kDa) and β (90–110 kDa) subunits (Hynes, 1992). In the vertebrates, 18 α subunits and 8 β subunits form 24 αβ pairs (Barczyk *et al*., 2010; Hynes, 2002) (Fig. 1). Integrin families are separated into four major categories: those with specificity for intercellular adhesion molecules and inflammatory ligands (leukocyte integrins, α4, αE, αL, αM, αX, and αD), Arg-Gly-Asp (RGD) motifs (αIIb, αV, α5, and α8), collagens (α1, α2, α10, and α11), and laminins (α3, α6, and α7) (Campbell and Humphries, 2011; Humphries *et al*., 2006; Tolomelli *et al*., 2017). Both α and β subunits are type I transmembrane glycoproteins containing a relatively large extracellular domain (ectodomain), a single transmembrane domain, and a short cytoplasmic tail (Arnaout, 2016; Campbell and Humphries, 2011; Fan and Ley, 2015; Luo *et al*., 2007).

The ectodomain is an asymmetric structure with a “head” carrying two “legs” (~16 nm long). The head consists of a predicted seven-bladed β-propeller domain (~60 amino acids each) of an α subunit (Xiao *et al*., 2004; Xiong *et al*., 2001) (nine of eighteen α subunits also contain an additional ~200 amino acids αA/αI domain) (Larson *et al*., 1989) and a ~250 amino acid βA/βI-like domain inserted in a hybrid domain of β subunit. The αA/αI domain and βA/βI-like domain are homologous to small ligand-binding von Willebrand Factor type A (vWFA) domain (Arnaout, 2002; Arnaout *et al*., 2007). The βA/βI-like domain contains two additional segments: one forms the interface with the β-propeller, and the other is a specificity-determining loop (SDL) mediating the ligand-binding (Luo *et al*., 2007). As structures of αVβ3 and αIIbβ3 showed, the α subunit leg domain is composed of an immunoglobulin-like “thigh” domain, a genu loop, and two similar β-sandwich domains named calf-1 and calf-2. The β subunit leg is formed by a plexin-semaphorin-integrin (PSI) domain, a hybrid domain (Bork *et al*., 1999), four tandem epidermal growth factor (EGF)-like domains, and a β-tail domain (βTD) (Bode *et al*., 1988; Janowski *et al*., 2001). The knee of the α subunit (α genu) lies at the junction between the thigh and calf-1 domains, and the knee of the β-subunit (β genu) is within the PSI and EGF1–2 region (Takagi and Springer, 2002). In integrins containing an αA/αI domain, ligand binding is mediated by this domain. As for integrins lacking the αA/αI domain, binding sites of ligands localize in the interface between β subunit β-I domain and α subunit β-propeller domain. Transmembrane domains of both α and β subunits are single α-helixes. NMR studies of αIIbβ3 show that the transmembrane domain of β3 is longer than αIIb and tilted with a ~25° angle to ensure the formation of inner and outer membrane clasp (IMC and OMC), which are important for proper integrin activity (Ginsberg, 2014; Kim *et al*., 2011; Lau *et al*., 2009; Sun *et al*., 2018).

### Conformations of integrins

Many techniques have been applied to distinguish two major models of conformational changes influencing integrin affinity, namely “switchblade” (Luo *et al*., 2007) and “deadbolt” (Arnaout *et al*., 2005). Although height change is a conspicuous readout, no consistent conclusions have been drawn owing to the plasticity of integrin structure. Most studies of ectodomains favor the switchblade model: extension (E^+^) of the integrin is the prerequisite for rearrangement of the ligand-binding site, leading to high affinity (H^+^). Three major conformations with different ligand binding affinities provide evidence for this model: inactive bent ectodomain with low-affinity headpiece (E^−^H^−^), primed extended ectodomain with low-affinity headpiece (E^+^H^−^) with low affinity, and fully activated extended ectodomain with high-affinity headpiece (E^+^H^+^) (Chen *et al*., 2010; Springer and Dustin, 2012; Takagi *et al*., 2002). However, crystallography results showed that the conformations of bent ectodomain with open headpiece (E^−^H^+^) found in αvβ3 and αXβ2 (Sen *et al*., 2013) had the capacity to bind its ligand. In primary human neutrophils, the “switchblade” transition (E^−^H^−^ to E^+^H^−^ to E^+^H^+^) was observed. And an alternative transition from E^−^H^−^ to E^−^H^+^ to E^+^H^+^ was also observed (Fan *et al*., 2016). E^−^H^+^ β2 integrins bind intercellular adhesion molecules (ICAMs) in cis (Fan *et al*., 2016) and form a face-to-face orientation (Fan *et al*., 2019), inhibiting leukocyte adhesion and aggregation (Fan *et al*., 2016). E^−^H^+^ αMβ2 integrins were shown binding FcγRIIA in cis to limit antibody-mediated neutrophil recruitment (Saggu *et al*., 2018). These findings suggest an alternative allosteric pathway other than the “switchblade” model.

### Integrin labeling in fluorescence imaging

#### Monoclonal antibodies

Immunofluorescent staining is the most commonly used method for integrin labeling, and antibody selection is extremely important for studying integrins. Monoclonal antibodies targeting different epitopes of specific integrin α and β subunits have been developed (Tab. 1). Some of these have been discussed in a previous review (Byron *et al*., 2009). Briefly, most of these clones target human integrins and can be classified into three categories: blocking/inhibitory, non-blocking/non-functional, and stimulatory/activation specific. Blocking antibodies can be used in integrin loss-of-function assays, such as adhesion and phagocytosis, or testing integrin expression when there is no ligand binding, such as flow cytometry. Non-blocking antibodies do not interfere with the biological functions of integrins. Thus, they are useful in live-cell fluorescence imaging to monitor the expression, localization, and clustering of integrins when interacting with ligands (Ezratty *et al*., 2009; Garmy-Susini *et al*., 2013; Huang *et al*., 2009; Jamerson *et al*., 2012; Shao *et al*., 2019; Tchaicha *et al*., 2011; Xiao *et al*., 2019). Among integrin antibodies, a unique kind of integrin antibody recognizes epitopes only expressed when integrins are activated or inactivated. Some of them further stabilize certain conformation(s) by steric effect resulting in enhancement or attenuation of ligand binding. Immunofluorescent imaging using antibodies with different effects on integrin activation can help illuminate novel biological functions.

Integrin antibodies that recognize activated epitopes have been applied to understanding β2 integrins-leukocyte-specific integrins that are critical for leukocyte recruitment and functions. Monoclonal antibody KIM127 (Robinson *et al*., 1992) recognizes the cysteine-rich repeat residues in the stalk region of integrin β2 subunits (Lu *et al*., 2001a). Monoclonal antibody mAb24 (Dransfield and Hogg, 1989) recognizes Glu173 and Glu175 within the CPNKEKEC sequence (residues 169–176) of the β2 I domain (Kamata *et al*., 2002; Lu *et al*., 2001b). These epitopes are shielded by the stalk region, and the αA/αI domain or the β-propeller of integrin α subunit are exposed and recognized by KIM127 and mAb24 upon integrin activation. KIM127 binding indicates integrin extension (E^+^), and mAb24 binding indicates rearrangement in the ligand-binding site leading to high-affinity (H^+^) (Kuwano *et al*., 2010; Lefort *et al*., 2012; Sorio *et al*., 2016). Owing to noninterference with each other (Fan *et al*., 2016), KIM127 and mAb24 were used to label different conformational states of β2 integrin on live human neutrophils (Fan *et al*., 2019; Fan *et al*., 2016; Sun *et al*., 2020a; Wen *et al*., 2020b), which enables to distinguish E^+^H^−,^ E^−^H^+,^ and E^+^H^+^ β2 integrins in live cells and. These studies demonstrated that other than the canonical switchblade model (E^−^H^−^ to E^+^H^−^ to E^+^H^+^), an alternative integrin activation pathway (E^−^H^−^ to E^−^H^+^ to E^+^H^+^) exists on primary human neutrophils. Monoclonal antibody 327C has been mapped to the upstream C-terminal region between amino acids 23 and 411 of the β2 integrin and also reports β2 integrin H^+^ (Zhang *et al*., 2008). 327C has been used to monitor β2 integrin activation during neutrophil migration (Green *et al*., 2006) and T cell spreading (Feigelson *et al*., 2010) using epifluorescence imaging, and neutrophil-platelet interaction using confocal microscopy (Evangelista *et al*., 2007).

Antibodies for activated integrins have also been used to study β1 integrins, which are expressed on various cells, such as leukocytes (Rullo *et al*., 2012; Werr *et al*., 1998), endothelial cells (Xanthis *et al*., 2019), epithelial cells (Spiess *et al*., 2018), and fibroblasts (Samarelli *et al*., 2020), and they are critical for several cell functions, such as adhesion and migration. Monoclonal antibody 9EG7 binds to the upper portion of the lower β-leg, which is approximately within the I-EGF2 domain, and reports β1 integrin extension (Lenter *et al*., 1993; Su *et al*., 2016) similar to KIM127 binding in β2 integrin. Antibody 12G10 binds to the βI domain of high-affinity β1 integrin (Su *et al*., 2016), which is similar to mAb24 binding in β2 integrin. Using 9EG7, 12G10, and a pan-β1 integrin antibody AIIB2, distinct nanoclusters of active and inactive β1 integrins have been identified in focal adhesions (FAs) (Spiess *et al*., 2018). Antibody TS2/16 binds an epitope similar to what 12G10 binds, where it activates and appears to stabilize an H^+^ βI domain conformation without requiring extension or hybrid domain swing-out (Van De Wiel-Van Kemenade *et al*., 1992). Antibodies HUTS-4, HUTS-7, and HUTS-21 recognize overlapping epitopes located in the hybrid domains of the β1 subunit. Their expressions parallel the ligand-binding activity of β1 integrins induced by various extracellular and intracellular stimuli (Luque *et al*., 1996; Su *et al*., 2016).

Antibodies recognizing and binding to the inactive conformation or that inhibit function are also used for integrin labeling. mAb13 recognizes an epitope within the βI domain of β1 integrin and is dramatically attenuated in the ligand-occupied form of α5β1. The binding of mAb13 to ligand-occupied α5β1 induces a conformational change in the integrin, resulting in the displacement of the ligand (Mould *et al*., 1996). Antibody SG/19 has been reported to inhibit the function of the β1 integrin on the cell surface. SG/19 recognizes the wild-type β1 subunit that exists in a conformational equilibrium between the high and low-affinity states but binds poorly to a mutant β1 integrin that is locked in a high-affinity state. SG/19 binds Thr82 located at the outer face of the boundary between the I-like and hybrid domains of the β1 subunit. SG/19 attenuates the ligand-binding function by restricting the conformational shift to the high-affinity state involving the swing-out of the hybrid domain without directly interfering with ligand docking (Luo *et al*., 2004). Monoclonal antibody SNAKA51 binds to the calf-1/calf-2 domains of the α5 subunit when the α5β1 integrin is active (Su *et al*., 2016). Alexa Fluor 488-conjugated SNAKA51 facilitates the detection of a conformation that promotes fibrillar adhesion formation. Gated stimulated emission depletion (g-STED) confocal microscopy analyses of PPFIA1 (protein tyrosine phosphatase receptor type F polypeptide interacting protein α1) and SNAKA51 activating α5β1 integrin in endothelial cells indicates that PPFIA1 localizes close to both focal and fibrillar adhesions (Mana *et al*., 2016).

β3 integrins are also widely expressed, and antibodies have been developed to study their functions. Vitronectin receptor integrin αVβ3 is expressed on leukocytes (Antonov *et al*., 2011), endothelial cells (Liao *et al*., 2017), and platelets (Bagi *et al*., 2019), etc. Active and inactive conformations of αVβ3 integrins can be detected by antibodies anti-αVβ3 clone LM609 and clone CBL544, respectively (Drake *et al*., 1995). WOW-1 is a ligand-mimic Fab fragment that reports αVβ3 integrin activation (Pampori *et al*., 1999). It has been used in detecting αVβ3 integrin activation on endothelial cells during shear sensing (Tzima *et al*., 2001) and migration (Lu *et al*., 2006) using fluorescence imaging. αIIbβ3 integrins are also known as glycoprotein IIb/IIIa and expressed on platelets (Adair *et al*., 2020; Chen *et al*., 2019; Ting *et al*., 2019). Antibody MBC370.2 binds to the calf-1 domain of the αIIb chain and reports the E+ of αIIbβ3 integrins (Zhang *et al*., 2013). PAC-1 is a ligand-mimic antibody and binds to both the β-propeller and βA/βI-like domains of H^+^ αIIbβ3 integrins (Kashiwagi *et al*., 1997). AP5 recognizes an epitope in the β3 PSI domain and reports hybrid domain swing-out (Cheng *et al*., 2013). By using these three antibodies, it has been demonstrated that biomechanical platelet aggregation is mediated by E^+^ but not H^+^ of αIIbβ3 integrins (Chen *et al*., 2019).

Integrin α4β7 is a lymphocyte homing receptor that mediates both rolling and firm adhesion of lymphocytes on vascular endothelium, two of the critical steps in lymphocyte migration and tissue-specific homing (Berlin *et al*., 1993; Iwata *et al*., 2004). Integrin α4β7 is the target of the most successful integrin drug vedolizumab, which is a human-derived blocking antibody and has recently proven useful in the treatment of inflammatory bowel diseases (Fedyk *et al*., 2012; Ley *et al*., 2016; Sands *et al.*, 2019; Zingone *et al*., 2020). An activation-specific antibody J19 for integrin α4β7 has been developed (Qi *et al*., 2012). This antibody does not block the mucosal vascular addressin cell adhesion molecule 1 (MAdCAM-1) binding site. Its binding site has been mapped to Ser-331, Ala-332, and Ala-333 of the β7 A/I-like domain and a seven-residue segment from 184 to 190 of the α4 β-propeller domain.

#### Fluorescent proteins

Since the molecular cloning of green fluorescent protein (GFP) from the jellyfish *Aequorea victoria* (Chalfie *et al*., 1994; Prasher *et al*., 1992; Ward *et al*., 1980), a wide spectrum of fluorescent proteins have provided excellent opportunities to monitor integrin localization and dynamics in living cells and tissues.

To study the separation of integrin α and β “legs” during activation, the monomeric cyan fluorescent protein (mCFP) and monomeric yellow fluorescent protein (mYFP) were fused to the C-termini of the α and β cytoplasmic domains of αVβ3, respectively (Kim *et al*., 2003). The “leg” separation was demonstrated by the decrease of Förster resonance energy transfer (FRET) from mCFP to mYFP. A similar strategy has been applied to study αMβ2 integrin activation as well (Lefort *et al*., 2009). To extend this idea in studying integrin activation in mouse disease models, knock-in (KI) mice with αM-mYFP (Lim *et al*., 2015), αL-mYFP (Capece *et al*., 2017), or β2-mCFP (Hyun *et al*., 2012) were generated, in which the fluorescent proteins were inserted into the C terminus of each integrin. Intravital imaging was then performed to visualize αM-mYFP^+^ leukocytes (Lim *et al*., 2015) or β2-mCFP leukocytes (Hyun *et al*., 2012) within inflamed or infected tissues. The αL-mYFP KI mice helped reveal an intracellular pool of integrin αLβ2 involved in CD8^+^ T cell activation and differentiation (Capece *et al*., 2017). In combined KI mice, activation of αLβ2 and αMβ2 was observed during neutrophil transendothelial migration by intravital microscopy (IVM) (Hyun *et al*., 2019).

In another study, GFP was inserted into the β3-β4 loop of blade 4 of the αL integrin β-propeller domain with no appreciable influence on integrin function and conformational regulation (Nordenfelt *et al*., 2017). The orientation of GFP can be measured by emission anisotropy microscopy (Ghosh *et al*., 2012; Nordenfelt *et al*., 2017; Ojha *et al*., 2020). Thus, they found that the direction of actin flow dictates integrin αLβ2 orientation during leukocyte migration (Nordenfelt *et al*., 2017). The role of α5 integrins in cell adhesion and migration was investigated by introducing the eukaryotic expression vectors pEGFP-N3, pECFP-N1, and pEYFP-N1 inserted with the integrin α5 cDNA and a 10–13 amino acid linker into CHO K1 and CHO B2 (α5-deficient) cells (Laukaitis *et al*., 2001). They found that α5 integrins stabilized cell adhesion and formed visible complexes after the arrival of α-actinin and paxillin. Integrin β4-YFP fusion proteins were introduced into HaCat cells as a marker of hemidesmosome protein complexes (HPCs). Meanwhile, CFP-tagged α-actinin was used as a marker of focal contacts (FCs). Tight co-regulation of HPCs and FCs was detected in keratinocytes undergoing migration during wound healing (Ozawa *et al*., 2010). Wild type or mutated mouse integrin β3-EGFP fusion protein was used to investigate the mechanisms and dynamics of the clustering and incorporation of activated αVβ3 integrins into FAs in living cells. Formation of the ternary complex consisting of activated integrins, immobilized ligands, talin, and PI(4,5)P_2_ was found to contribute to integrin clustering (Cluzel *et al*., 2005). Fluoppi is a technology providing an easy way to visualize protein-protein interactions (PPIs) with a high signal-to-background ratio (Koyano *et al*., 2014; Yamano *et al*., 2015). It employs an oligomeric assembly helper tag (Ash-tag) and a tetrameric fluorescent protein tag (FP-tag) to create detectable fluorescent foci when there are interactions between two proteins fused to the tags. This technique has been used to prove the interaction of integrin β1 and Procollagen-Lysine, 2-Oxoglutarate 5-Dioxygenase 2 (PLOD2) in cell migration (Ueki *et al*., 2020).

In another study, an extracellular site of integrin β1 was reported suitable for inserting different tags, including GFP and PH-sensitive pHluorin (Huet-Calderwood *et al*., 2017). pHluorin is a GFP variant that displays a bimodal excitation spectrum with peaks at 395 and 475 nm and an emission maximum at 509 nm. Upon acidification, pHluorin excitation at 395 nm decreases with a corresponding increase in the excitation at 475 nm (Mahon, 2011). In this study, pHluorin tagged integrin β1 was used to monitor the exocytosis of β1 integrins in live cells. Since similar extracellular fluorescence protein insertion was performed in β2 integrins (Bonasio *et al*., 2007; Moore *et al*., 2018; Nordenfelt *et al*., 2017), it is feasible to use pHluorin in study β2 integrin functions, such as degranulation and phagocytosis.

#### Other methods for fluorescently tagging integrins

HaloTag is a 34 kDa engineered, catalytically inactive derivative of a bacterial hydrolase. It can be fused to a protein of interest and covalently bound by synthetic HaloTag ligands with high specificity. A covalent bond can form rapidly under physiological conditions and is essentially irreversible. HaloTag allows adaptation of the targeted protein to different experimental requirements without altering the genetic construct (Los *et al*., 2008; Los and Wood, 2007). For example, Atto655 was used to generate the HaloTag655 ligand, which is suitable for labeling live cells by expressing a β1-integrin-HaloTag fusion protein. The resulting living cells are suitable for STED microscopy, and intracellular distribution of the β1-integrin such as filopodia and endocytic vesicles were studied in unprecedented detail (Schroder *et al*., 2009). Halo and SNAP tags were also inserted into the β1 integrin extracellular domain in the study mentioned above (Huet-Calderwood *et al*., 2017). Similar to HaloTag, SNAP (Keppler *et al*., 2003) is also a self-labeling protein tag that can covalently bind to synthetic fluorescence dyes. Sequential fluorescence dye labeling of Halo-tagged integrin β1 can distinguish surface and internal β1 integrins in cells (Huet-Calderwood *et al*., 2017).

Many integrins bind to ECM molecules through an RGD motif. RGD peptide was found to bind to resting integrins and induce integrin activation. Compared to linear peptides, suitable optimized cyclic RGD (cRGD) peptides interact with integrins in a more selective manner and with higher affinity (Weide *et al*., 2007). Changing a three-dimensional structure or modifying the amino acid sequences flanking the RGD motif can enhance its ligand selectivity (Schaffner and Dard, 2003). Within this area, integrin αVβ3 was studied most extensively for its role in tumor growth, progression, and angiogenesis. It was considered an interesting biological target for therapeutic cancer drugs and a diagnostic molecular imaging probe (Ye and Chen, 2011). Fluorescein isothiocyanate (FITC)-conjugated dimeric cRGD peptides (FITC-RGD2, FITC-3P-RGD2, and FITC-GalactoRGD2) were used as fluorescent probes for *in vitro* assays of integrin αvβ3/αvβ5 expression in tumor tissues (Zheng *et al*., 2014). Quantum dots (QDs) are fluorescent nanocrystals that absorb a wide-range spectrum (400–650 nm) of light and emit a narrow symmetric spectrum of bright fluorescence. These allow the QD signal to be clearly distinguished from the cellular autofluorescence background (Alivisatos *et al*., 2005; Gao *et al*., 2005; Michalet *et al*., 2005; Pinaud *et al*., 2006). cRGD peptides and a biotin-streptavidin linkage are used to specifically couple individual QDs to αVβ3 integrins on living osteoblast cells. The positions of individual QDs were tracked with nanometer precision, and localized diffusive behavior was observed (Lieleg *et al*., 2007). Near-infrared (650–900 nm) fluorescence imaging has provided an effective solution for improving the imaging depth along with sensitivity and specificity by minimizing the autofluorescence of some endogenous absorbers (Shah and Weissleder, 2005; Tung, 2004). Cyanine analogs, such as Cy5, Cy5.5, were used to label cyclic RGD analogs for *in vivo* optical imaging of integrin αVβ3 positive tumors with high contrast in mice (Jin *et al*., 2006; Wang *et al*., 2004).

The C-terminal region of the fibrinogen γ subunit contains γC peptide uniquely binding to activated or primed αIIbβ3 integrin at the interface between α and β subunits (Hantgan *et al*., 2006; Springer *et al*., 2008; Zhao *et al*., 2016). Therefore, it may serve as the prototype for the design of a probe targeting activated αIIbβ3 integrin. Gold nanoclusters are a newly developed class of fluorescent particles. The gold nanocluster Au_18_ conjugated with γC peptide peptides were used to detect αIIbβ3 in HEL with an excitation wavelength of 514 nm and an emission wavelength of 650 nm (Zhao *et al*., 2016). Due to the specific binding between the Leu-Asp-Val (LDV) peptide and integrin α4β1, fluorophore-conjugated LDV is commonly used to monitor changes of α4β1 integrin conformation or affinity in live cells (Chigaev *et al*., 2001; Chigaev *et al*., 2011b). LDV-FITC can be used as a FRET donor to reveal conformational changes of α4β1 under different biological conditions (Chigaev *et al*., 2003b; Chigaev *et al*., 2004; Njus *et al*., 2009).

Soluble ligands ICAM-1 (Lefort *et al*., 2012; Margraf *et al*., 2020), vascular cell adhesion protein 1 (VCAM-1) (Sun *et al*., 2014), and MadCAM-1 (Sun *et al*., 2018; Sun *et al*., 2014) were used to detect the activation of β2, β1, and β7 integrins. In the classic article imaging the immunological synapse (Grakoui *et al*., 1999), Cy5-labeled ICAM-1 were anchored to the bilayer in a manner that allows their free diffusion in the supported bilayer to monitor the dynamic changes of integrin αLβ2 activation and distribution during the formation of the immunological synapse. A similar approach became a canonical method to study integrins in immunological synapses (Kaizuka *et al*., 2007; Kondo *et al*., 2017; Somersalo *et al*., 2004) and was also used to track active integrin αLβ2 in leukocyte migration (Smith *et al*., 2005).

Fluorophore-conjugated integrin allosteric antagonists and agonists are also widely used to label certain integrins. BIRT 377 and XVA-143 are integrin αLβ2-specific allosteric antagonists that belong to two distinct classes. The BIRT 377 binding site is located within the I domain of the αL integrin subunit. The XVA-143 site is located between the αL β-propeller and the β2 subunit I-like domain (Shimaoka and Springer, 2003). BIRT- and XVA-FITC were used to study conformational changes of integrin αLβ2 (Chigaev *et al*., 2015). A ligand-mimic small molecular probe has been developed to measure integrin αLβ2 activation (Chigaev *et al*., 2011a).

### Imaging techniques

#### Live-cell imaging of integrins

Live-cell imaging has been abundantly used in biological studies, including some for integrins. This method has given rise to tremendous progress in documenting dynamic cellular processes, such as cell adhesion (Fan *et al*., 2016; Morikis *et al*., 2017; Morikis *et al*., 2020; Shao *et al*., 2020; Sun *et al*., 2020a; Sun *et al*., 2018; Wen *et al*., 2020b; Yago *et al*., 2015; Yago *et al*., 2018), migration (Kostelnik *et al*., 2019; Moore *et al*., 2018; Nordenfelt *et al*., 2017; Panicker *et al*., 2020; Ramadass *et al*., 2019; Tweedy *et al*., 2020; Zhou *et al*., 2020), cell-cell interactions (Hanna *et al*., 2019; Kretschmer *et al*., 2019; Lin *et al*., 2015a; Lin *et al*., 2015b; Omsland *et al*., 2018; Zucchetti *et al*., 2019), endocytosis/phagocytosis (Chu *et al*., 2020; Freeman *et al*., 2020; Levin-Konigsberg *et al*., 2019; Ostrowski *et al*., 2019; Walpole *et al*., 2020), exocytosis/degranulation (Cohen *et al*., 2015; Thiam *et al*., 2020), and cytoskeleton rearrangement (Balint *et al*., 2013; Ostrowski *et al*., 2019; Walpole *et al*., 2020), in real-time and down to the single molecular level (Balint *et al*., 2013; Katz *et al*., 2017; Katz *et al*., 2019; Moore *et al*., 2018; Mylvaganam *et al*., 2020). Fluorescent probes and proteins have been ubiquitously utilized in live-cell imaging, allowing observation of dynamics and function of cellular structures and macromolecules, such as integrins, over time and in-depth.

In epifluorescence microscopy, which is the most commonly used wide-field microscopy, all the emission light around the focal plane captured by the objective, which depends on its numerical aperture, is sent to the detector leading to high light-collecting efficiency. The use of the pinhole in confocal laser scanning microscopy (CLSM) decreases the background signal from out-of-focus light and increases the signal-to-background ratio. However, CLSM is limited by phototoxicity/photobleaching. This is mainly due to that most confocal microscopes have detectors with low quantum efficiency, such as photomultiplier tubes (PMT), in comparison to epifluorescence microscopes, such as charge-coupled device (CCD) or complementary metal-oxide-semiconductor (CMOS) cameras. Thus, to acquire images of similar brightness, CLSM needs higher power of the excitation light than epifluorescence microscopy. On the other hand, most CLSM setting has a limited imaging speed due to its scanner. For example, most CLSM has a laser dwell time of ≥1 μs per pixel (Straub *et al*., 2020), which means that it will take more than 0.25 seconds to acquire a 512 × 512 image (≤4 frames per second). In comparison, most cameras in epifluorescence microscopes allow an imaging speed of ≥20 frames per second (1280 × 1024 pixels). The low speed of CLSM can be overcome by using a high-cost resonant scanner, which allows a speed of 30 fps for 512 × 512 images. Thus, if the specimen is a monolayer, epifluorescence microscopy might be a good choice (Stephens and Allan, 2003; Waters, 2007).

Epifluorescence microscopy has been used to monitor β2 integrin activation during leukocyte rolling on selectins (Kuwano *et al*., 2010). In the study developing the integrin αL-mYFP mice, an intracellular pool of αL integrins was discovered in CD8+ T cells using epifluorescence microscopy (Capece *et al*., 2017). In the study of the integrin αM-mYFP mice, epifluorescence images showed that αM integrins enriched in the lamellipodia during neutrophil migration (Lim *et al*., 2015). Epifluorescence-based live-cell fluorescence lifetime imaging microscopy (FLIM)-FRET has been used to demonstrate the cis interaction between sialylated FcγRIIA and the αI-domain of integrin αMβ2 (Saggu *et al*., 2018). In another study, epifluorescence imaging of platelet integrin αIIbβ3 showed that biomechanical platelet aggregation in disturbed flow is mediated by E^+^H^−^ αIIbβ3 integrins (Chen *et al*., 2019).

For thicker (e.g., 20–100 μm) live-cell specimens, CLSM was used for imaging integrins (Fan *et al*., 2019; Lin *et al*., 2015a; Lock *et al*., 2018; Sahgal *et al*., 2019; Schymeinsky *et al*., 2009). For example, the distribution of integrin αLβ2 during immunological synapse formation was visualized using CLSM (Lin *et al*., 2015a). Imaging by CLSM, Integrin αVβ5 was found to forms novel talin- and vinculin-negative reticular adhesion structures, which may be required for mediating attachment during mitosis (Lock *et al*., 2018). CLSM was also used to investigate the recycling of active β1 integrins regulated by GGA2 and RAB13 (Sahgal *et al*., 2019). CLSM imaging of β2 integrins illustrated the role of mAbp1 in regulating β2 integrin-mediated phagocytosis and adhesion (Schymeinsky *et al*., 2009). CLSM helped to show the distribution of active β2 integrins during lymphocyte migration, and roles of talin, ZAP-70, rap2, and SHARPIN during lymphocyte migration (Evans *et al*., 2011; Pouwels *et al*., 2013; Smith *et al*., 2005; Stanley *et al*., 2008; Stanley *et al*., 2012)

However, the slower imaging speed and higher phototoxicity limit its usage for live-cell imaging. There are some implementations that significantly increase imaging speed and reduce phototoxicity under the condition of CLSM. Such implementations include slit scanning and pinhole multiplexing methods, including spinning disk confocal microscopy (SDCM) (Graf *et al*., 2005; Maddox *et al*., 2003). In addition to the fundamental disk containing thousands of pinholes in a spiral, there is a second collector disk with a matching pattern of microlenses focusing excitation light with up to 70% efficiency onto the imaging pinholes. In combination with an electron-multiplying charge-coupled device (CCD) detector, SDCM turns to be an ideal solution for fast live-cell confocal imaging of thicker specimens (Wang *et al*., 2005). Using SDCM, it was found that ADP-ribosylation factor 6 directs the traffic of α9 and β1 integrins on dorsal root ganglion neurons (Eva *et al*., 2012). The dynamic changes of β5 integrins were visualized by SDCM during mitosis, which suggested that a selective role for integrin β5 in mitotic cell attachment (Lock *et al*., 2018). In another study, it was found that phosphatidylinositol 3,4,5-trisphosphate binder Rasa3 was translocated to integrin αIIbβ3 and involved in the integrin outside-in signaling on platelets during α-thrombin stimulation (Battram *et al*., 2017).

Another high-resolution live-cell imaging technique is total internal reflection fluorescence (TIRF) microscopy. In TIRF microscopy, a laser incident beam illuminating the boundary between two media of different refractive indices (usually the coverslip and the specimen) experiences total internal reflection. The totally internally reflected laser beam generates the evanescent wave, which excites fluorophores that are in the vicinity of the coverslip-specimen interface (~100–200 nm), resulting in a very high signal-to-background image with a ~100 nm optical section compared to ~700 nm of confocal or wide-field (Axelrod, 1981, 2001; Hocde *et al*., 2009). The high signal-to-background is at the cost of penetration. TIRF can only reveal structures close to the coverslip surface, such as membrane proteins and FAs. As a family of membrane proteins, integrin molecules are highly suitable for analysis with TIRF microscopy. Almost all integrin molecules have been monitored by TIRF microscopy. By using TIRF, it has been shown that FA disassembly during cell migration requires endocytosis of β1 integrins, which is regulated by clathrin (Chao and Kunz, 2009). TIRF imaging also showed that mechanical stimuli disassemble β1 integrin clusters and enhance endocytosis of integrins expressed on human umbilical vein endothelial cells (HUVECs) (Kiyoshima *et al*., 2011). H^+^ β2 integrins reported by monoclonal antibody 327C have been imaged by TIRF microscopy during neutrophil arrest and demonstrated that H^+^ β2 integrin-ICAM-1 binding initiates calcium influx (Dixit *et al*., 2011), and kindlin-3 is responsible for β2 integrin H^+^ (Dixit *et al*., 2012). H^+^ β2 integrins can also be reported by mAb24 (Dransfield and Hogg, 1989; Kamata *et al*., 2002; Lu *et al*., 2001b), as mentioned before. By using TIRF microscopy, the H^+^ β2 integrins were found polarized to the lead-edge during T cell migration (Hornung *et al*., 2020). It has also been demonstrated that β2 integrins form podosomes of dendritic cells imaged by TIRF microscopy (Gawden-Bone *et al*., 2014). In another study, a Rap1-GTP-interacting adapter molecule (RIAM)/lamellipodin-talin-integrin (β3) complex that guides cell migration was discovered by using TIRF microscopy (Lagarrigue *et al*., 2015). The transport of β3 to FA has been imaged by TIRF microscopy and was found to be regulated by an AAK1L- and EHD3-dependent rapid-recycling pathway (Waxmonsky and Conner, 2013). The PDK1-mediated endocytosis of β3 integrin during FA disassembly has also been monitored by TIRF microscopy (Di Blasio *et al*., 2015).

**FIGURE 2.**
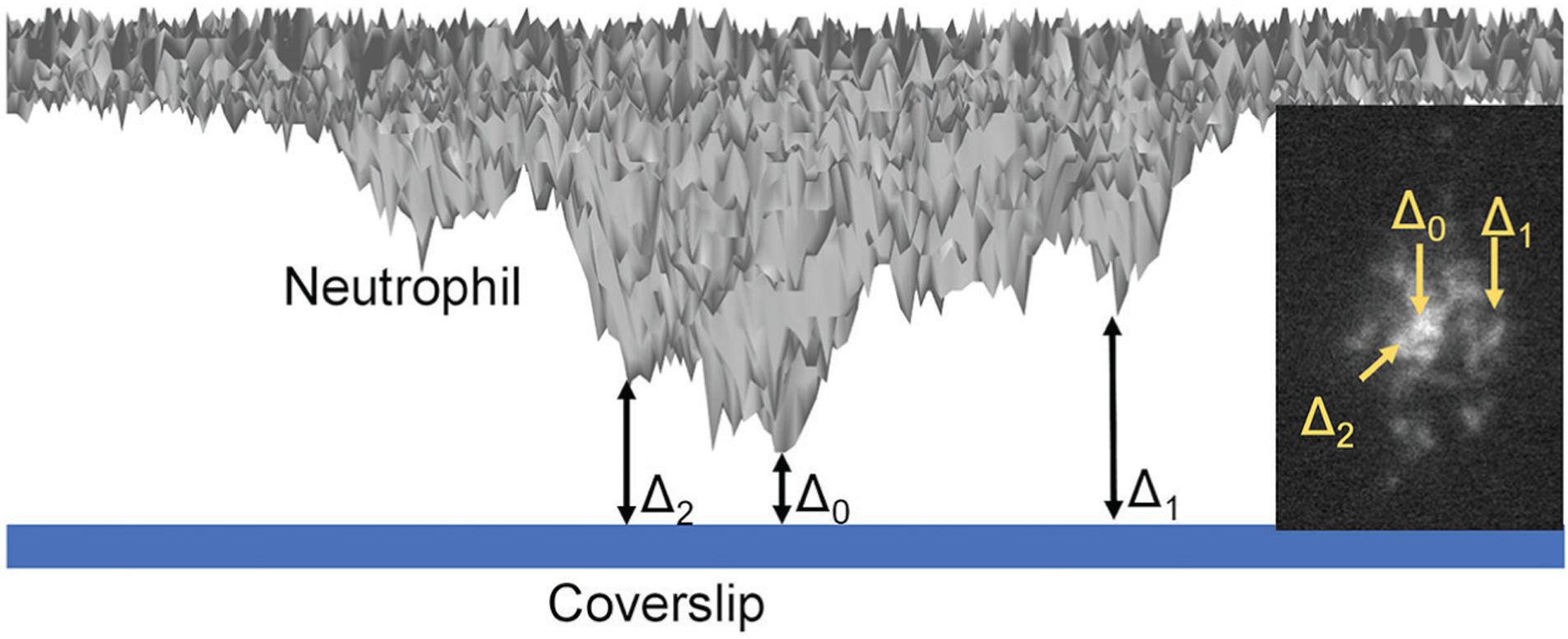
Schematics of qDF (quantitative dynamic footprinting) microscopy. The side-view neutrophil footprint (~100 nm) converted from the TIRF (total internal reflection fluorescence) membrane fluorescence image (inset image) was shown (grey surface). The distance of the closest approach of the neutrophil with the coverslip is Δ_0_. This is the position with the brightness cell-membrane fluorescence signal (shown in the inset image). The z-distance (Δ) of other positions was calculated by their cell-membrane fluorescence signal. Two examples (Δ_1_ and Δ_2_) were shown.

As an update to TIRF microscopy, quantitative dynamic footprinting (qDF) microscopy was developed in 2010 (Sundd *et al*., 2010), based on the calculation of the evanescent wave intensity and the fluorescence signals of the cell membrane. In the development of qDF microscopy, a two-step calibration procedure involved: (1) The distance of the closest approach of a stationary neutrophil with the coverslip was measured using variable angle TIRF microscopy and was designated Δ_0_ (Suppl. Fig. 3 in Sundd *et al*. (2010)); and (2) The z-distance (Δ) of any region in the neutrophil footprint is calculated by fluorescence intensity using the following equation, Δ = Δ_0_ + λ/4π × (n_1_^2^ × sin^2^ θ − n_2_^2^)^−1/2^ × ln (I_Fmax_(θ)/I_F_(θ)). Fig. 2 described the Δ_0_ and Δ (Two examples Δ_1_ and Δ_2_ are shown). In this equation, λ is the wavelength of the emission light, and n_1_ and n_2_ are the refractive indexes of the two medium types, such as glass coverslip and cell, respectively. qDF microscopy was used to reveal neutrophil rolling under high shear stress (Sundd *et al*., 2010; Sundd and Ley, 2013) and was used in monitoring the dynamics of β2 integrin activation during human neutrophil arrest (Fan *et al*., 2019; Fan *et al*., 2016). By combining qDF with conformational reporting antibodies KIM127 (Lu *et al*., 2001a; Robinson *et al*., 1992) and mAb24 (Dransfield and Hogg, 1989; Kamata *et al*., 2002; Lu *et al*., 2001b), the canonical switchblade model of β2 integrin activation (Luo *et al*., 2007) was confirmed (Fan *et al*., 2016). Meanwhile, an unexpected E^−^H^+^ conformation of β2 integrins was observed, which suggested an alternative pathway of β2 integrin activation that E^−^H^−^ integrins can acquire high-affinity first (E^−^H^+^) and then extended (E^+^H^+^). The E^−^H^+^ β2 integrins can bind ICAM ligands expressed on the same neutrophil in cis and inhibit integrin activation and neutrophil adhesion (Fan *et al*., 2016).

#### Super-resolution imaging of integrins

The spatial resolution of microscopic techniques is limited by Abbe’s law, according to which the highest achievable lateral and axial resolution (d_x,y_ and d_z_), or diffraction limits, can be:

$${\text{d}}_{\text{x},\text{y}}=\frac{\lambda }{2\text{NA}}$$

$${\text{d}}_{\text{z}}=\frac{2\lambda }{{\text{NA}}^{2}}$$

in which λ is the wavelength of the excitation beam, and NA is the numerical aperture of the microscope objective. NA = n sinα, with n being the refractive index of the medium and α being the half-cone angle of the focused light produced by the objective (Abbe, 1881; Hon, 1882). For example, in a conventional microscope, when a specimen is excited by blue-green light whose wavelength is about 488–550 nm, and an oil immersion objective with NA = 1.40 is used, lateral and axial resolution can be ~200 nm and ~500 nm, respectively (Thompson *et al*., 2002). Abbe’s law holds only true for wide-field microscopes.

Several super-resolution techniques circumvent the limits of diffraction and increase both lateral and axial resolution. One approach beyond the limit of diffraction is to sharpen the point-spread function of the microscope by spatially patterned excitation, including STED (Hell and Wichmann, 1994; Klar *et al*., 2000), reversible saturable optically linear fluorescence transitions (RESOLFT) (Hell, 2003, 2007, 2009; Hofmann *et al*., 2005), structured-illumination microscopy (SIM) (Gustafsson, 2000), and saturated structured-illumination microscopy (SSIM) (Gustafsson, 2005). Another is a pointillist approach that requires localization of individual fluorescent molecules (single-molecule localization microscopy, SMLM), such as stochastic optical reconstruction microscopy (STORM) (Rust *et al*., 2006), photoactivated localization microscopy (PALM) (Betzig *et al*., 2006), fluorescence photoactivation localization microscopy (fPALM) (Hess *et al*., 2006), points accumulation for imaging in nanoscale topography (PAINT) (Sharonov and Hochstrasser, 2006), ground-state depletion (GSD) microscopy (Folling *et al*., 2008). Expansion microscopy (ExM) expands the sample using a polymer system. Positions of labeled molecules were measured by using conventional microscopes. Based on the factor of expansion, the localization of these molecules in the unexpanded cells can be calculated back to achieve nanoscale resolution (Chen *et al*., 2015). Several super-resolution microscopy techniques have been summarized before (Galbraith and Galbraith, 2011; Pujals *et al*., 2019; Wen *et al*., 2020a), but some will be described here in more detail (Tab. 2).

Super-resolution imaging techniques have been used to study integrin molecules in recent years. Interferometric photoactivation and localization microscopy (iPALM) was used to visualize the three-dimensional structure of FAs, which includes the integrin αV and paxillin-enriched integrin signaling layer, the talin and vinculin-enriched force transduction layer, and zyxin and vasodilator-stimulated phosphoprotein-enriched actin regulatory layer (Kanchanawong *et al*., 2010). SIM was used to illustrate the linear β1 integrin distribution in FAs (Hu *et al*., 2015). Using a new super-resolution imaging technique with a similar principle to PALM, signal molecular tracking of β1 and β3 integrin molecules was performed, and they were found entering and exiting from FAs and repeatedly exhibiting temporary immobilizations (Tsunoyama *et al*., 2018). Using both STED and STORM microscopy, both active and inactive β1 integrins were visualized in FAs and were found segregating into distinct nanoclusters (Spiess *et al*., 2018). STED was also used in testing the colocalization of active α5β1 integrins and PPFIA1 to demonstrate the role of PPFIA1 in active α5β1 integrin recycling. In another study, both active β1 and β5 integrins were found separately located in FAs (Stubb *et al*., 2019) by Airyscan confocal microscopy, a super-resolution technique with similar resolution compared to SIM (Huff, 2015). Airyscan confocal microscopy utilized a 32-channel gallium arsenide phosphide photomultiplier tube (GaAsP-PMT) area detector that collects a pinhole-plane image at every scan position. Each detector element functions as a single, very small pinhole. Knowledge about the beam path and the spatial distribution of each detector channel enables very light-efficient imaging with improved resolution and signal-to-noise ratio. αV and β5 integrins in FAs were also imaged by iPALM in this study. Airyscan confocal microscopy was also used to identify the colocalization of GGA2, RAB13, and active β1-integrins to demonstrate the role of GGA2 and RAB13 in β1-integrin recycling (Sahgal *et al*., 2019), and image the localization of α11 and β1 integrins on mammary gland stromal fibroblast spreading on collagen (Lerche *et al*., 2020). GSD microscopy was used to visualize the LPS-induced colocalization of chloride intracellular channel protein 4 (CLIC4) and β1 integrins, demonstrating the role of CLIC4 in cell adhesion and β1 integrin trafficking (Argenzio *et al*., 2014). By using iPALM, the extension of αLβ2 integrins was monitored by the axial movement of the αLβ2 headpiece towards the coating substrate during Jurkat T cell migration (Moore *et al*., 2018). Using Fab fragments of mAb24 and KIM127, the distribution of E^−^H^+^, E^+^H^−^, and E^+^H^+^ β2 integrins on neutrophil footprint during arrest was visualized by STORM (Fan *et al*., 2019). Combined with molecular modeling, the SuperSTORM technique was developed (Fan *et al*., 2020), and the orientation of E^−^H^+^, E^+^H^−^, and E^+^H^+^ β2 integrins were indicated. This work enabled visualizing integrin molecules at the single molecular level and was the first to show the orientation of different conformation integrins. An unexpected face-to-face orientation of E^−^H^+^ β2 integrins is held by cis interaction with ICAM dimers (Fan *et al*., 2019). Airyscan confocal microscopy was used in imaging β2 integrin activation on neutrophils interacting with HUVECs (Fan *et al*., 2019). Our work (Fan *et al*., 2019) and a previous one (Moore *et al*., 2018) mentioned above were both focusing on the conformational changes of β2 integrins. Using iPALM, Moore *et al*. (2018) were able to show the E^+^ of β2 integrins by measuring the distance of β2 integrin headpiece to the substrate. In our work, we measured not only the E^+^ but also the H^+^ of β2 integrins. We can report all three active β2 integrin conformations (E^−^H^+^, E^+^H^−^, and E^+^H^+^). The pitfall of our work is that we assessed fixed samples, and iPALM can assess live cells. STED was used to show the colocalization of integrin αLβ2 and low-density lipoprotein receptor-related protein 1 (LRP1) on neutrophils during cytokine midkine-induced neutrophil recruitment. (Weckbach *et al*., 2019). PALM was used to identify integrin β3 nanoclusters within FAs (Deschout *et al*., 2016; Deschout *et al*., 2017) and discover the role of integrin β3 nanoclusters in bridging thin matrix fibers and forming cell-matrix adhesions (Changede *et al*., 2019).

#### Intravital imaging of integrins

Whereas cellular behavior is different between *in vitro* and *in vivo* settings, biological processes are the sum of individual cellular behaviors shaped by many environmental factors. Endless efforts have been made to image cells residing in live animals at microscopic resolution, giving rise to intravital microscopy (IVM), an ever-developing field. In its infancy, blood flow within microvessels and circulating leukocytes targeting to inflamed tissue have been seen through bright field transillumination (Kunkel *et al*., 2000; Ley *et al*., 1993; Pittet and Weissleder, 2011; Ramadass *et al*., 2019). With the advent of fluorescence microscopy, genetically encoded fluorescent proteins (Cappenberg *et al*., 2019; Deppermann *et al*., 2020; Girbl *et al*., 2018; Honda *et al*., 2020; Hsu *et al*., 2019; Lammermann *et al*., 2013; Lefort *et al*., 2012; Marcovecchio *et al*., 2020; Matlung *et al*., 2018; McArdle *et al*., 2019; Owen-Woods *et al*., 2020; Powell *et al*., 2018; Schleicher *et al*., 2000; Uderhardt *et al*., 2019; Wen *et al*., 2020b; Wolf *et al*., 2018) and fluorescent dyes staining cells *ex vivo* before adoptive transfer or injected directly into the animal to enable visualization of endogenous structures are now available (Arokiasamy *et al*., 2019; Bousso and Robey, 2004; Deppermann *et al*., 2020; Girbl *et al*., 2018; Honda *et al*., 2020; Marcovecchio *et al*., 2020; Marki *et al*., 2018; Owen-Woods *et al*., 2020; Rapp *et al*., 2019; Schoen *et al*., 2019; Uderhardt *et al*., 2019; Vats *et al*., 2020; Wen *et al*., 2020b; Wolf *et al*., 2018). Detection of responses of individual cells within their natural environment over extended periods of time and space thus has become possible.

Epifluorescence microscopy can be used as IVM for studying integrins. One study showed that after 24 h of cecal ligation puncture, β1 integrins were found in the neutrophil extracellular traps in the liver and helped to sequester circulating tumor cells (Najmeh *et al*., 2017). In another study, RGD-Quantum Dot was used to report integrin activation on tumor vessel endothelium (Smith *et al*., 2008). Confocal microscopes can also be used for IVM. Spinning disk confocal IVM was used to visualize β3 integrins expressed on vascular endothelial cells, which tethers and interacts with *Borrelia burgdorferi* in circulation during infection (Kumar *et al*., 2015). Integrin α2 has been used as a marker for platelet aggregates in the spinning disk confocal intravital imaging of hepatic ischemia-reperfusion injury (Van Golen *et al*., 2015). Multiphoton laser scanning microscopy is another popular method for IVM. Its conception is based on the principle that a fluorophore can not only be excited by one high-energy photon but also two simultaneous low-energy near-infrared photons with longer wavelengths of around 700 to 1,000 nm (Göppert-Mayer, 2009; Kawakami *et al*., 1999). Two-photon excitation needs a very high local photon density, which is reached at the focal plane. Thus, only fluorophores in the focal plane can be excited in two-photon microscopy. Fluorophores outside the focal plane are highly unlikely to be excited, making a high signal-to-background ratio. In confocal microscopy, fluorophores outside the focal plane will also be exited. In comparison, two-photon microscopy will have less photobleaching of fluorophores outside the focal plane, resulting in the lowest phototoxicity possible (Squirrell *et al*., 1999; Svoboda and Block, 1994). Great improvement of penetration depths (200–300 μm or even 1000 μm) and longer recording periods can be achieved by this technology (Benninger and Piston, 2013; Hickman *et al*., 2009; Kobat *et al*., 2011; Theer *et al*., 2003). Thus, multiphoton microscopy is a great choice of intravital imaging.

As mentioned before, integrin β2-mCFP mice were developed (Hyun *et al*., 2012), and these mice helped discover a β2 integrin-enriched uropod elongation during leukocyte extravasation using multiphoton IVM. Integrin αM-mYFP mice were developed (Lim *et al*., 2015) as well. In this study, the migration of αM+ leukocytes in the cremaster or trachea during fMLP stimulation or influenza infection was imaged by multiphoton IVM, respectively. In the follow-up study using αM-mYFP/β2-mCFP and αL-mYFP/β2-mCFP mice (Hyun *et al*., 2019), the activation of integrin αMβ2 and αLβ2 were reported by FRET *in vivo* for the first time using multiphoton IVM. It was found that αLβ2 is more important than αMβ2 in neutrophil transendothelial migration.

#### Förster Resonance Energy Transfer (FRET) of integrins

Since there are large conformational changes during integrin activation, techniques sensitive to distance changes like FRET become useful tools in studying integrins. FRET used as a “molecular ruler” ushered in the quantification of intermolecular interactions (Johnson, 2005; Stryer and Haugland, 1967). The concept of FRET was originally proposed by Teodor Förster in 1948. FRET is a phenomenon of quantum mechanics involving two matched fluorophores when the emission spectrum of the donor fluorophore overlaps with the excitation spectrum of the acceptor fluorophore. When the two fluorophores are in close physical juxtaposition (≤10 nm), the excitation of the donor results in emitted photons, which are quenched by and transfer the energy to the acceptor, resulting in the emission of acceptor fluorescence (Huebsch and Mooney, 2007; Periasamy, 2001). The efficiency of energy transfer is inversely related to the 6th power of the inter-molecular distance:

$$\text{E}=\frac{1}{1+{\left(\text{r}/{\text{R}}_{0}\right)}^{6}}$$

E is the efficiency, r is the intermolecular distance, and R_0_, known as Förster constant, is the value of r when this pair of donor and acceptor achieve 50% FRET efficiency. R_0_ depends on the overlap integral of the donor emission spectrum with the acceptor absorption spectrum and their mutual molecular orientation as expressed by the following equation:

$${\text{R}}_{0}=\frac{9\left(\text{ln}10\right)}{128\pi^{5}{\text{N}}_{\text{A}}}\cdot \frac{{\text{Q}}_{\text{D}}\kappa^{2}}{\eta^{4}}\cdot \text{J}$$

in which N_A_ is Avogadro’s number; Q_D_ is the fluorescence quantum yield of the donor in the absence of acceptor; κ^2^. is the dipole orientation factor; η is the refractive index of the medium; and J.is the spectral overlap integral of the donor-acceptor pair (Wang and Chien, 2007). Therefore, the range over which FRET can be observed is very narrow; only intra- and inter-molecular distances within ~2–10 nm can be detected (Huebsch and Mooney, 2007; Periasamy, 2001). The FRET efficiency can be altered by any change of the orientation or distance between the two fluorophores (Tsien, 1998).

To obtain a FRET signal for studying the interaction of two proteins, they must be fluorescently labeled. One approach is to label the antibodies or antagonist/agonist binding to the two proteins with proper fluorophores. Fluorophore-conjugated antagonist/agonist can be synthesized, while labeling kits facilitating covalent binding (usually using amide bonds) of many different fluorescent molecules to antibodies are commercially available (Fan *et al*., 2019; Fan *et al*., 2016; Masi *et al*., 2010; Wen *et al*., 2020b). Another approach is introducing genes of two fluorescent proteins (FPs) to the donor/acceptor pair of proteins, respectively. Owing to their excellent extinction coefficients, quantum yield, and photostability, cyan fluorescence protein (CFP) and yellow fluorescence protein (YFP) are the most commonly used pair for FRET (Giepmans *et al*., 2006; Tsien, 1998). Green fluorescence protein (GFP) and red fluorescence protein (RFP) can also be utilized as a pair of fluorophores for FRET (Bajar *et al*., 2016; Lam *et al*., 2012). Genetic manipulation is conducted to gain recombinant fused genes, and the 1:1 ratio of donor/adaptor protein to CFP/YFP greatly simplifies the calculations of FRET efficiency and the quantification of protein interactions. One drawback of fusion proteins is the possibility to exhibit altered biological function or molecular structure. Thus, careful characterization before FRET is recommended (Masi *et al*., 2010; McArdle *et al*., 2016).

Measurements of (1) signal intensity and (2) fluorescence lifetimes are two major ways to determine FRET efficiency. Regarding the signal intensity method, the comparable changes between the intensification of the acceptor’s emission and synchronous decrease in donor’s emission facilitate the detection of FRET by splitting the emission from the two fluorophores. The split lights are then filtered through a specific filter set and collected separately. The downsides of this method are: (1) the excitation light of acceptor may excite the donor owing to the possible overlap of their excitation spectrum, (2) the leak of donor emission to the detecting channel of the acceptor, and (3) the faster photobleaching of the donor compared with that of the acceptor (Masi *et al*., 2010). The fluorescence lifetime is an intrinsic property of fluorophores. It is the characteristic time that a fluorophore stays in the excited state before the emission of the fluorescence photon. Fluorescence lifetime imaging microscopy (FLIM) uses pulsed excitation lasers to acquire quantitative information through measurements of fluorescence lifetimes (Lakowicz *et al*., 1992; Le Marois and Suhling, 2017). Based on the fact that fluorescence lifetime decreases proportionally with the efficiency of FRET, FLIM-FRET serves as a precise way to determine FRET efficiency (Suhling *et al*., 2015). Although spectral overlap must always be taken into consideration in both methods, FLIM can rule out the influence of local fluorophore concentration or fluorescence intensity leading to the defects in signal intensity measurement (Lakowicz and Masters, 2008). There are additional strategies to measure FRET efficiency. “Donor de-quenching” (or “Acceptor photo-bleach”) method photo-bleaches the acceptor; thus, the increase of fluorescence in the “de-quenched” donor is proportional to FRET efficiency. FRET efficiency can be determined by measurement of donor fluorescence intensity before and after photobleaching of the acceptor. This method is an endpoint measurement making it incompatible with dynamic monitoring (Carman, 2012; Periasamy, 2001; Wang and Chien, 2007).

**FIGURE 3.**
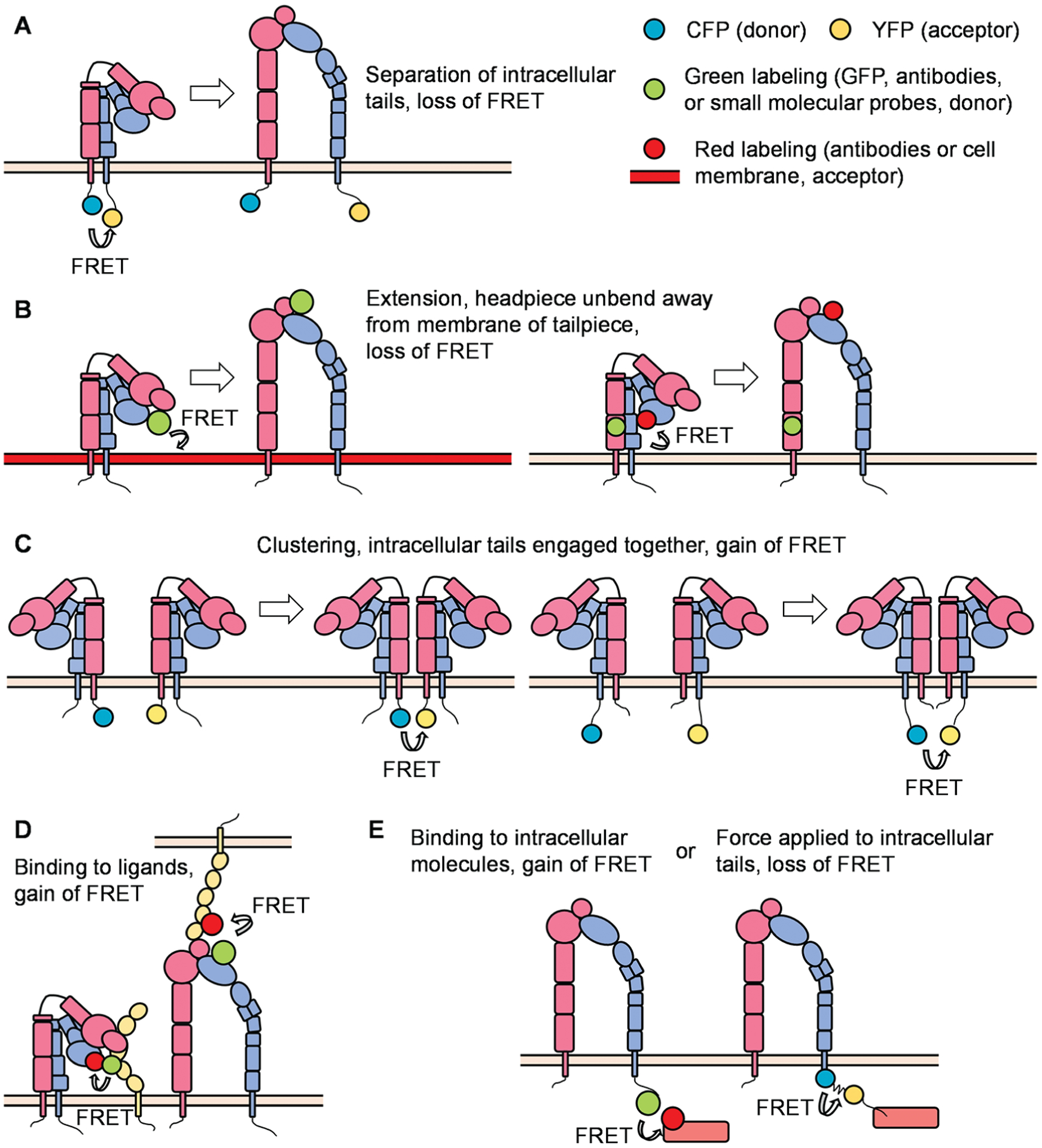
Principles of FRET (Förster resonance energy transfer) in integrin studies. (A) The cytoplasmic tails of α and β subunits were labeled with FRET donor and acceptor, respectively. The separation of cytoplasmic tails is assessed by the reduction of FRET. (B) The integrin headpiece and cell membrane/integrin tailpiece were labeled with FRET donor and acceptor, respectively. The extension/unbent of integrin ectodomain is assessed by the reduction of FRET. (C) The cytoplasmic tails of α or β subunits were labeled with both FRET donor and acceptor. The clustering of integrin molecules is assessed by the increase of FRET. (D–E) The interaction of integrins and their ligands (D, both in cis and in trans) or cytoplasmic regulators (E, interaction or force measurement) can be assessed by FRET.

With the help of the improvement in microscopic techniques and labeling with fluorophores, great advantages have been made regarding integrin conformation and signaling. FRET can be used to identify the spatial movement of integrin cytoplasmic tails (Fig. 3A). In a classical study, leukocytes were stably transfected with FRET donor and acceptor pair mCFP and mYFP at the C-termini of the integrin αL and β2 subunits, respectively. In the resting state, high FRET efficiency was measured, indicating that the c-termini of the αL and β2 subunits were close to each other. Upon the triggering of the integrin inside-out signaling (chemokine SDF-1 and its receptor CXCR4) or outside-in signaling (ICAM-1 in the presence of Mn^2+^), the FRET efficiency was significantly reduced, indicating a spatial separation of αL and β2 cytoplasmic tails. Bidirectional integrin signaling is accomplished by coupling extracellular conformational changes to the separation of the cytoplasmic domains (Kim *et al*., 2003). A similar strategy has been applied to study αMβ2 integrin activation (αM-mCFP, β2-mYFP) as well (Fu *et al*., 2006; Lefort *et al*., 2009). The first dual-fluorescent protein KI mice - αLβ2 FRET (αL-YFP/ β2-CFP) mice and αMβ2 FRET (αL-YFP/ β2-CFP) mice - have been successfully constructed. By using two-photon intravital ratiometric analysis of (CFP/YFP) in neutrophils from these mice, determination of differential regulation of integrin αLβ2 and αMβ2 during neutrophil extravasation became realized (Hyun *et al*., 2019).

FRET can also be used to identify conformational changes in the integrin ectodomain domains. One method is to label the integrin headpiece and cell membrane/integrin tailpiece with FRET donor and acceptor, respectively, to measure the extension/unbending of integrins (Fig. 3B). In some studies, the LDV-FITC probe binding to the α4-integrin headgroup and octadecyl rhodamine B incorporated into the plasma membrane were used as the donor/acceptor pair for FRET assays. Several publications have proved the feasibility of detecting the extension of integrin α4β1 (Chigaev *et al*., 2003a; Chigaev *et al*., 2008; Sambrano *et al*., 2018). Integrin αIIbβ3 at the surface of blood platelets plays a primary role in hemostasis. FRET using fluorescently labeled Fab fragments of monoclonal antibodies targeting the βA/I-like domain of β3 subunit (donor, Alexa Fluor 488 conjugated P97 Fab) and the calf-2 domain of αIIb subunit (acceptor, Cy3-M3 Fab or Cy3-M10 Fab) can determine the distance between these two domains at rest (about 6 nm) or activation (about 17 nm) states. Researchers found that activated αIIbβ3 in living platelets exhibits a conformation less extended than proposed by the switchblade model (Coutinho *et al*., 2007). In another study, a FITC-conjugated monoclonal antibody against integrin αM headpiece and octadecyl rhodamine B incorporated into the plasma membrane were used as the donor/acceptor pair for FRET assays to measure the extension of integrin αMβ2 (Lefort *et al*., 2009). Two distinct allosteric antagonists (BIRT 377 and XVA-143) targeting the αLI domain and β2 subunit I-like domain were used as donors. FRET conducted on live cells using a real-time flow cytometry approach was used to measure the distance between these two donors and a novel lipid acceptor PKH 26. Researchers found that triggering of the pathway used for T-cell activation (phorbol ester and thapsigargin) induced rapid extension of the integrin αLβ2 (Chigaev *et al*., 2015).

Instead of attaching donor and acceptor respectively to α and β subunits, studying integrin micro-clustering requires attachment of both the donor and acceptor to either the α or β subunit within one heterodimeric integrin (Fig. 3C). In this case, integrin micro-clustering will lead to FRET. In a study focused on *Drosophila* α_PS2C_β_PS_ integrin, mVenus and mCherry were fused to cytoplasmic and transmembrane domains of integrin β subunits. Mutations in α subunit cytoplastic domain (GFFNR to GFANA) or β subunit (V409D), which showed higher affinity for ligands, showed ~2–3-fold higher FRET values compared to that of wild type (Smith *et al*., 2007). In another study, K562 cells were transiently transfected with αL-mCFP, αL-mYFP, and wild-type β2, generating approximately equal amounts of αL-mCFP/2 and αL-mYFP/2 cells. The binding of ICAM-1 oligomers resulted in significant micro-clustering. In contrast, monomeric ICAM-1 did not induce integrin αLβ2 clustering (Kim *et al*., 2004). Using the same methodology, researchers found the disruption of the αLβ2 transmembrane domain by mutation of a key interface residue Thr-686 in the β2 transmembrane domain promoted binding of αLβ2 with ICAMs and facilitated αL microcluster formation (Vararattanavech *et al*., 2009).

FRET can also be used to assess interactions of the integrin headpiece with its ligands (Fig. 3D) and integrin cytoplasmic domains with the cytoskeleton and various signaling molecules (Fig. 3E) during integrin inside-out and outside-in signaling. In our previous study, we used FRET to detect the in-cis interaction of E^−^H^+^ β2 integrins and ICAM-1 (Fan *et al*., 2016). HA58-FITC, which binds ICAM-1 domain 1 and blocks its interaction with integrin αLβ2, but not integrin αMβ2, was used as the FRET donor. Antibody mAb24-DyLight 550 binding β2 integrin H^+^ headpiece was used as the acceptor. When integrin αMβ2 bound ICAM-1 in cis, the two antibodies were close enough to have FRET. When this interaction was blocked by mAb R6.5, which binds to integrin αMβ2-binding domain 3 of ICAM-1, or replacing the acceptor by KIM127- DyLight 550 (binding to the knees of E^+^ β2 integrins), FRET did not occur. These results indicate that E^−^H^+^ integrin αMβ2 binds ICAM-1 in cis (Fan *et al*., 2016). In another study, antibodies against FcγRIIA (Alexa Fluor 488) and integrin αMβ2 (Alexa Fluor 568) were used as donor and acceptor, respectively, to demonstrate the cis interaction of integrin αMβ2 and FcγRIIA by FLIM-FRET (Saggu *et al*., 2018). High-throughput dynamic three-color single molecule-FRET tracking was conceived. Orthogonal labeling of RGD and PHSRN motifs within fibronectin serve as FRET donor (Alexa Fluor 555) and acceptor (Alexa Fluor 594) at residues 1381 and 1500, respectively. FRET signatures are distinctive for the folded and unfolded state. The extracellular domain of αvβ3 was labeled with Alexa Fluor 647. By monitoring the intensity of all three dyes, the impact of fibronectin conformation and dynamics on αvβ3 integrin-binding can be determined. A more stable fibronectin-αvβ3 complex was observed when fibronectin exhibited a more folded conformation (Kastantin *et al*., 2017). Interaction of PKCα with β1 integrin was detected by FLIM-FRET performed in MCF7 cells, in which GFP-PKCα fusion protein was used as the donor, and integrin β1 antibody conjugated with Cy3.5 was used as the acceptor (Ng *et al*., 1999). Using FLIM-FRET, GFP-conjugated β1 integrin of mouse embryonic fibroblasts was found to interact with mRFP conjugates of the talin rod domain and α-actinin but not the talin head domain or paxillin (Parsons *et al*., 2008). Schwartz and colleagues have constructed a FRET-based tension sensor methodology, which consists of monomeric teal fluorescent protein (mTFP1) and monomeric Venus (mVenus) joined by a 40 amino-acid elastic linker (Faulon Marruecos *et al*., 2016). The elastic linker can elongate upon tensile force in the range of 0–6 pN. Incorporation of this reporter into the β2 subunit of integrin αLβ2 enabled researchers to find that actin polymerization and extracellular ligand-binding are in a positive feedback loop (Nordenfelt *et al*., 2016). FRET was used to assess the association of β1-integrin and ErbB2, which is an important integrator of transmembrane signaling by the EGFR family, on tumor cells. (Mocanu *et al*., 2005).

## Conclusions

Overall, optical imaging of integrin molecules helps us understand the regulation of integrin expression, localization, clustering, conformational changes, and functions. Although there are various antibodies targeting integrin to visualize integrins with different conformations, most of these antibodies are specific for human integrin molecules. This limits the use of these antibodies for studying integrins in physiologically-relevant *in vivo* systems, such as mouse disease models, as well as in loss-of-function assays of integrin regulators because it is impossible to do genetic editing in humans. It has been reported that introducing human β2 integrins restores the infectious deficiency in β2 integrin knockout mice (Wilson *et al*., 1993). Thus, replacing the mouse integrin gene with human integrin cDNA might be a way to expand the use of existing integrin antibodies.

As we discussed, super-resolution microscopy is a powerful tool for studying integrins. However, their uses in integrin studies are mostly restricted to phenomenon reports and morphology studies. Thus, finding a way to dig into the molecular details of integrin regulation and function using super-resolution microscopy needs more attention. For example, super-resolution imaging can better assess the clustering of integrin molecules. Assessing the localization of important integrin modulators, such as talin, kindlin, RIAM, etc., by super-resolution microscopy will help understand their roles in regulating integrin activation.

FRET is a powerful tool to study dynamic changes in integrin conformation, but most FRET assays of integrins are restricted in cell lines. Only two integrin FRET mouse strains (αLβ2 and αMβ2) were developed. Thus, the development of more integrin FRET mouse strains is needed to visualize integrin conformation changes *in vivo*. Those mice could also be used in studying molecular mechanisms of integrin regulation and functions or in different disease models.

Although many techniques were developed to visualize integrin molecules as we reviewed above, whether the fluorescence labeling affects integrin function needs to be demonstrated in the specific studies, especially for activating specific integrin antibodies and fluorescent protein tags. For example, KIM127 was reported to stimulate leukocyte aggregation (Robinson *et al*., 1992), and mAb24 may lock the H+ conformation of β2 integrins (Smith *et al*., 2005). Thus, when using them in imaging, whether they affect the specific function interested in your study becomes critical. When we use them in studying integrin activation during neutrophil rolling and arrest, we tested that they do not affect ligand binding of β2 integrins and neutrophil arrest (Fan *et al*., 2016). This is the same case for fluorescent protein tags. In the iPALM study of β2 integrin (Moore *et al*., 2018), a mEos3.2 tag was inserted in the β-propeller domain of the αL-subunit of integrin αLβ2. They measure the axial movement of the mEos3.2 tag to report E+ of integrin αLβ2. They have tested that the fluorescence protein insertion in this site does not affect cell adhesion and ICAM-1 binding (Bonasio *et al*., 2007). In another study, a CFP-YFP tension sensor was inserted into the β2 integrin cytoplasmic tail to measure the force bearing of β2 integrins during cell migration using FRET (Nordenfelt *et al*., 2016). They have demonstrated that the insertion they used does not affect cell migration compared to cells transfected with wild-type β2 integrins.

## Figures and Tables

**TABLE 1:** Human integrin-targeting monoclonal antibodies

Integrin	Epitope (Domain)	Clone Name	Integrin	Epitope (Domain)	Clone Name
**Blocking/inhibitory**					
α1	αA/αI	FB12 (Fabbri *et al*., 1996)	β1	βA/βI-like	4B4 (Takada and Puzon, 1993)
					mAb13 (Takada and Puzon, 1993)
α2	αA/αI	12F1 (Kamata *et al*., 1994)			AIIB2 (Takada and Puzon, 1993)
		Gi9 (Tuckwell *et al*., 2000)			P4C10 (Takada and Puzon, 1993)
		JA218 (Tuckwell *et al*., 2000)		Hybrid	JB1A (Ni and Wilkins, 1998)
		P1E6 (Kamata *et al*., 1994)			
			β2	βA/βI-like	CLB LFA-1/1 (Zang *et al*., 2000)
α3	β-propeller	ASC-6 (Zhang *et al*., 1999)			MHM23 (Hildreth *et al*., 1983)
		P1B5 (Zhang *et al*., 1999)			TS1/18 (Lu *et al*., 2001a)
	Not known	IA3 (Turner *et al*., 2006)			IB4 (Wright *et al*., 1983)
					L130 (Zang *et al*., 2000)
α4	β-propeller	HP2/1 (Kamata *et al*., 1995)		Hybrid	7E4 (Tng *et al*., 2004)
		P4C2 (Kamata *et al*., 1995)			
		PS/2 (Kamata *et al*., 1995)	β3	βA/βI-like	7E3 (Artoni *et al*., 2004)
	Not known	9F10 (Lei *et al*., 2016)		Not known	SZ-21 (Sheng *et al*., 2003)
		L25 (Chandele *et al*., 2016)			
		P1H4 (Stampolidis *et al*., 2015)	β4	Not known	ASC-8 (Egles *et al*., 2010)
		A4-PUJ1 (Martin *et al*., 2015)			
			β5	Not known	ALULA (Su *et al*., 2007)
α5	β-propeller	JBS5 (Burrows *et al*., 1999)			
		mAb16 (Burrows *et al*., 1999)	β6	Not known	6.3G6 (Weinreb *et al*., 2004)
		P1D6 (Burrows *et al*., 1999)			
	Not known	NKI-SAM-1 (Orecchia *et al*., 2003)	β7	βA/βI-like	FIB504 (Andrew *et al*., 1994)
					FIB27 (Andrew *et al*., 1994)
					FIB30 (Andrew *et al*., 1994)
α6	Not known	GoH3 (Lee *et al*., 1992)			
			β8	Not known	37E1 (Mu *et al*., 2002)
α7	Not known	6A11 (Zhao *et al*., 2004)			
			αIIb	β-propeller	10E5 (Nishimichi *et al*., 2015)
α8	β-propeller	YZ3 (Nishimichi *et al*., 2015)			2G12 (Kamata *et al*., 1996)
α9	Not known	Y9A2 (Staniszewska *et al*., 2008)	αVβ3	β-propeller	23C6 (Kamata *et al*., 2013)
			αVβ5	Not known	P1F6 (Singh *et al*., 2007)
αV	β-propeller	17E6 (Kamata *et al*., 2013)			P3G2 (Hendey *et al*., 1996)
		L230 (Nishimichi *et al*., 2015)			
	Not known	NKI-M9 (Grzeszkiewicz *et al*., 2001)	αVβ6	Not known	10D5 (Weinreb *et al*., 2004) 6.3G9 (Weinreb *et al*., 2004)
αE	αA/αI	αE7-1 (Russell *et al*., 1994)			
		αE7-2 (Russell *et al*., 1994)	αLβ2	αA/αI, β-propeller, and βA/βI-like	YTA-1 (Zang *et al*., 2000)
	Not known	Ber-ACT8 (Kruschwitz *et al*., 1991)			
αL	αA/αI	TS1/22 (Lu *et al*., 2004)	αM	αA/αI	2LPM19c (Osicka *et al*., 2015)
		HI111 (Ma *et al*., 2002)			MAN-1 (Eisenhardt *et al*., 2007)
		CBR LFA-1/1 (Ma *et al*., 2002)			anti-M7 (Wolf *et al*., 2018)
	Not known	mAb38 (Lomakina and Waugh, 2004)			ICRF44 (Osicka *et al*., 2015)
				Thigh	M1/70 (Osicka *et al*., 2015)
αX	αA/αI	3.9 (Hogg *et al*., 1986)	αD	αA/αI	217I (Van Der Vieren *et al*., 1999)
	Not known	496K (Sadhu *et al*., 2008)			240I (Van Der Vieren *et al*., 1999)
		Bu15 (Sadhu *et al*., 2008)			
**Non-blocking/non-functional**					
α1	Not known	TS2/7 (Woods *et al*., 1994)	α5	Calf-1 to 2	mAb11 (Askari *et al*., 2010)
				β-propeller	VC5 (Askari *et al*., 2010)
α2	Not known	16B4 (Tuckwell *et al*., 2000)			
		31H4 (Tuckwell *et al*., 1995)	α6	Not known	J1B5 (Damsky *et al*., 1994)
α3	Not known	A3-X8 (Weitzman *et al*., 1993)	α7	Not known	3C12 (Mielenz *et al*., 2001)
α4	Not known	44H6 (Bridges *et al*., 2005)	α9	Not known	A9A1 (Vlahakis *et al*., 2005)
		8F2 (Newham *et al*., 1998)			
αIIb	Not known	PL98DF6 (Puzon-Mclaughlin *et al*., 2000)	αV	Not known	LM142 (Mathias *et al*., 1998)
			αD	Not known	212D (Van Der Vieren *et al*., 1999)
αL	β-propeller	TS2/4 (Zang *et al*., 2000)			92C4D (Van Der Vieren *et al*., 1999)
	Not known	YTH81.5 (Stanley *et al*., 2008)			
αM	β-propeller	CBRM1/20 (Oxvig and Springer, 1998)	β1	I-EGF	K20 (Askari *et al*., 2010)
	Thigh	OKM1 (Osicka *et al*., 2015)	β2	Not known	CBR LFA-1/7 (Lu *et al*., 2001a)
		CyaA (Osicka *et al*., 2015)			
			β4	Not known	ASC-3 (Egles *et al*., 2010)
αX	Not known	CBR-p150/2E1 (Shang and Issekutz, 1998)			
			β5	Not known	11D1 (Ricono *et al*., 2009)
**Stimulatory or activation-specific**					
α2	Not known	JBS2 (Ho *et al*., 1997)	β2	βA/βI-like	mAb24 (Lu *et al*., 2001a)
					327C (Beals *et al*., 2001)
α4	β-propeller	HP1/3 (Kamata *et al*., 1995)		Hybrid	MEM-148 (Tang *et al*., 2005)
				EGF-like 2	KIM127 (Robinson *et al*., 1992)
α5	Calf-1 & 2	SNAKA51 (Clark *et al*., 2005)		EGF-like 3	CBR LFA-1/2 (Lu *et al*., 2001a)
					MEM-48 (Lu *et al*., 2001a)
αIIb	β-propeller	PT25-2 (Puzon-Mclaughlin *et al*., 2000)		EGF-like 4	KIM185 (Lu *et al*., 2001a)
	Calf-1	MBC370.2 (Chen *et al*., 2019)	β3	Hybrid	AP3 (Peterson *et al*., 2003)
	Calf-2	PMI-1 (Loftus *et al*., 1987)		PSI	AP5 (Cheng *et al*., 2013)
				EGF-like 3/4	LIBS6 (Frelinger *et al*., 1991)
αL	αA/αI	2E8 (Carreno *et al*., 2010)		β-tail	LIBS2 (Du *et al*., 1993)
		MEM83 (Gronholm *et al*., 2016)			
	Genu	NKI-L16 (Keizer *et al*., 1988)	β7	βA/βI-like and hybrid	10F8 (Tidswell *et al*., 1997)
					2B8 (Tidswell *et al*., 1997)
αM	αA/αI	CBRM1/5 (Oxvig *et al*., 1999)			2G3 (Tidswell *et al*., 1997)
	Thigh	VIM12 (Osicka *et al*., 2015)			
			αIIbβ3	β-propeller and βA/βI-like	PAC-1 (Kamata *et al*., 1996)
αX	Not known	496B (Sadhu *et al*., 2008)			
β1	βA/βI-like	12G10 (Mould *et al*., 1995)	αVβ3	β-propeller and βA/βI-like	WOW-1 (Pampori *et al*., 1999)
		8A2 (Takada and Puzon, 1993)			LM609 (Kamata *et al*., 2013)
		TS2/16 (Takada and Puzon, 1993)			
		A1A5 (Takada and Puzon, 1993)	αVβ6	β-propeller and βA/βI-like	6.8G6 (Weinreb *et al*., 2004)
	Hybrid	15/7 (Mould *et al*., 2003)			
		HUTS-4 (Luque *et al*., 1996)			
		HUTS-7 (Luque *et al*., 1996)	α4β7	β-propeller and βA/βI-like	J19 (Qi *et al*., 2012)
		HUTS-21 (Luque *et al*., 1996)			
	PSI	8E3 (Mould *et al*., 2005)			
		N29 (Mould *et al*., 2005)	β1	EGF-like 2	9EG7 (Askari *et al*., 2010)

**TABLE 2:** Claimed resolution of super-resolution microscopy used in integrin imaging

Name	lateral resolution	axial resolution
Structured-Illumination Microscopy	100 nm (Gustafsson *et al*., 2008)	250–350 nm (Gustafsson *et al*., 2008)
Airyscan Confocal Microscopy	120 nm (Huff *et al*., 2017)	350 nm (Huff *et al*., 2017)
Stimulated Emission Depletion Microscopy	45 nm (Neupane *et al*., 2014)	100 nm (Neupane *et al*., 2014)
Stochastic Optical Reconstruction Microscopy	20 nm (Rust *et al*., 2006)	50 nm (Huang *et al*., 2008)
Photoactivated Localization Microscopy	20 nm (Temprine *et al*., 2015)	50 nm (Temprine *et al*., 2015)
Interferometric Photoactivation and Localization Microscopy	20 nm (Shtengel *et al*., 2009)	10 nm (Shtengel *et al*., 2009)
Ground State Depletion Microscopy	20 nm (Dixon *et al*., 2017)	50 nm (Dixon *et al*., 2017)
